# Targeting Autophagy for Pituitary Tumors

**DOI:** 10.3390/cancers17091402

**Published:** 2025-04-23

**Authors:** Evan Yin, Motoyasu Satou, Toru Tateno

**Affiliations:** 1 Division of Endocrinology and Metabolism, Department of Medicine, University of Alberta, Edmonton, AB T6G 2G3, Canada; eyin1@ualberta.ca; 2 Department of Biochemistry, School of Medicine, Dokkyo Medical University, Tochigi 321-0293, Japan

**Keywords:** pituitary tumors, autophagy, cell proliferation, hormone production

## Abstract

Pituitary tumors, arising from the pituitary gland, can be classified as functioning or non-functioning based on their hormone production. Previous studies demonstrated that impairment of cellular processes, such as autophagy, a crucial cellular recycling mechanism, has been implicated in pituitary tumorigenesis and hormone dysregulation. This review comprehensively examines the intricate relationship between autophagy and pituitary tumors. We explore the multifaceted role of autophagy in cancer, highlighting its dual nature as both a tumor suppressor and a promoter depending on the context. We also discuss the specific mechanisms of autophagy, including macroautophagy, mitophagy, crinophagy, and their relevance to pituitary tumorigenesis and hormone regulation. Furthermore, we analyze the current literature regarding the impact of various therapeutic interventions in pituitary tumor cells, with both autophagy-promoting and autophagy-inhibiting strategies. We address the challenges in interpreting autophagy activity and its complex interplay with hormone production. Current evidence suggests the potential of targeting autophagy as a therapeutic approach for pituitary tumors, emphasizing further research and clinical trials to determine the optimal strategy for individual patients and improve long-term outcomes.

## Simple Summary

Pituitary tumors are abnormal growths on the pituitary gland, which regulates the body’s hormones. These tumors can disrupt hormone levels, causing various health issues. This review focuses on autophagy, a process where cells recycle and break down their components, and its role in pituitary tumors. Autophagy can either suppress or promote tumor growth, depending on the situation. The review explores different types of autophagy and their effects on tumor development and hormone imbalances. Researchers are investigating whether drugs that target autophagy could be used to treat pituitary tumors. Some drugs aim to boost autophagy, while others work to suppress it. However, measuring autophagy’s activity and understanding its relationship with hormone production is difficult. More research and clinical trials are needed to develop effective therapies that manipulate autophagy for treating pituitary tumors.

## 1. Introduction

Pituitary tumors (PTs) exhibit a significant prevalence within the general population, with estimates indicating a lifetime prevalence of approximately 17% [1,2] and constitute roughly 10% of all intracranial neoplasms [3]. PTs are abnormal growths in the pituitary gland and can cause several symptoms and complications due to uncontrolled tumor growth and hormonal imbalance. These tumors can be classified into functioning and nonfunctioning types depending on their ability to produce hormones. Functioning tumors can lead to various endocrine disorders, while non-functioning tumors may grow large enough to put pressure on surrounding structures. Autophagy, a cellular process that involves the degradation and recycling of damaged cellular components, plays a crucial role in maintaining cellular homeostasis and preventing the accumulation of dysfunctional proteins. Recent observations lead to the functional contribution of autophagy in pituitary tumor development and progression, as well as hormone dysregulation. Understanding these processes could provide valuable insights into novel therapeutic strategies for managing these tumors and improving patient outcomes.

## 2. Autophagy and Cancer

### 2.1. Crosstalk Between Autophagy and Cancer

Autophagy is an intracellular catabolic pathway that identifies and recycles excess or abnormal proteins, as well as damaged or excess cellular components, to maintain metabolic homeostasis [4]. Preservation of organelle function and signaling pathways via autophagy typically arises under cell stress conditions such as oxygen or nutrient deprivation, DNA damage, and anti-cancer therapies.

Most common instances of autophagy are classified as macroautophagy, in which a double-layered isolation membrane known as a phagophore develops from the endoplasmic reticulum and envelops targeted cargo to form an autophagosome [5]. The autophagosome then fuses with a lysosome and becomes an autolysosome, which degrades and recycles its cargo into basic components. Multiple points along this pathway are facilitated by a series of autophagy-related (ATG) proteins.

Development of the phagophore is initiated by the ULK complex, composed of UNC-51-like kinase 1 (ULK1), ULK2, focal adhesion kinase family interacting protein of 200 kD (FIP200), ATG13, and ATG101 [5]. Upon deactivation of the mechanistic target of rapamycin complex 1 (mTORC1) in low-nutrient conditions, ATG13 phosphorylation is inhibited, allowing the ULK complex to form. Likewise, low-nutrient conditions also activate AMP-activated protein kinase (AMPK), which promotes recruitment of ULK complex components and other downstream factors. The complex is then assisted by VPS34 complex I (comprising VPS34, beclin-1, ATG14, and VPS15), a PI3 kinase that plays roles in both autophagy and vesicle trafficking. VSP34 helps the phagophore membrane produce phosphatidylinositol-3-phosphate (PI3P). PI3P then expands the phagophore membrane and recruits the ATG16L1–ATG5–ATG12 complex, as well as ATG3 and ATG7, which lipidate ATG8 proteins embedded in the membrane [6]. VSP34 is essential for hormone secretory vesicles (SVs) budding from TGN [7,8,9]. Lipidated ATG8 proteins are responsible for most cases of autophagy, which are highly selective and arise when lipidated ATG8 proteins associate with autophagy cargo receptors (ACRs) that have recognized cargo marked for degradation via ubiquitination [10]. After the phagophore becomes an autophagosome and fuses with a lysosome, lysosomal hydrolases break down cargo into basic carbohydrates, lipids, and amino acids destined for other metabolic pathways.

A variety of regulatory factors are involved in autophagy with multiple modes of action. These include p53, a tumor suppressor that represses autophagy under normal conditions but promotes expression of AMPK and damage-regulated autophagy modulator 1 (DRAM1) under stress [4]. RAS activation or inactivation appears to promote autophagy, suggesting that its role depends on the cellular context.

Autophagy is an important process for preventing the development of malignancy in cells. Clearing any malfunctioning cellular components prevents the propagation of defective cellular function, DNA damage from reactive oxygen species (ROS), and incorrect antigen presentation [4]. Although autophagy plays an important part in tumor suppression, it can later be hijacked to promote survival and growth should a cell become cancerous, indicating it is a double-edged sword in cancer—a repressor in cancer initiation, then a promoter operating cancer proliferation and survival.

### 2.2. Autophagy as a Cancer Inhibitor

Autophagy contributes to tumor suppression by preventing the accumulation of cellular stress factors that can initiate tumorigenesis [11]. However, it is essential to recognize the context-dependent nature of autophagy’s role in cancer, as it can also promote survival in established tumors. Deficiencies in autophagy-related genes have been associated with increased susceptibility to tumorigenesis. For example, the Beclin 1 gene, a key regulator of autophagy, is frequently deleted from human cancers [12,13]. Certain factors upregulated in tumor cells, like p62/SQSTM1, play key roles in autophagy, inflammation, and/or oxidative stress response to prevent cell death [14]. Sustained p62 expression resulting from autophagy defects was sufficient to alter NF-κB regulation and gene expression and to promote tumorigenesis [14]. p62 is a target of selective autophagy; at the same time, p62-bound “substrates” are also degraded by autophagy. In this case, p62-dependent NFkB reduction causes tumorigenesis. In the same manner, p62 contributes to later tumorigenesis by interacting with kelch-like ECH-associated protein 1/nuclear factor erythroid 2-related factor 2 (Nrf2), tumor necrosis factor receptor-associated factor 6 (TRAF6), receptor-interacting serine/threonine-protein kinase-1 (RIPK1), mammalian mitogen-activated protein kinase (MAPK), and regulatory-associated protein of mTOR (RAPTOR) to protect cells from cell stress, allowing for greater cancer cell survival and growth as well as chemotherapy resistance [15,16]. Cancer cells exhibit elevated levels of p65 production, while decreased expression of p62 is observed in stromal cells. The therapeutic outcome may be influenced by strategies aimed at inactivating p62 [16].

Beclin-1 regulates and induces autophagy as a component of VPS34 complex I [5]. Certain factors interact with beclin-1 to inhibit its activity and thereby promote tumorigenesis through inhibition of autophagy, such as human epidermal growth factor receptor 2 (HER2), which was found to bind beclin-1 and prevent formation of VPS34 complex I, inducing tumorigenesis in breast cancer cells [17]. On the other hand, the abhydrolase domain containing 5 (ABHD5) was found to be an autophagy promoter and is important for binding to beclin-1 to prevent its inactivation by competitors such as CASP3 [18]. ABHD5 was found to play a role in suppressing the tumorigenesis of colorectal carcinoma by facilitating autophagy.

### 2.3. Mitophagy’s Dual Role in Cancer Growth over Time

Selective macroautophagy of mitochondria is known as mitophagy and plays both a tumor-suppressing and tumor-promoting role depending on the cell’s stage of cancer development [19]. When mitochondrial membrane potential drops beyond functional requirements, proteasomal degradation of PTEN-induced putative kinase 1 (PINK1) decreases, which causes PINK1 to accumulate in the mitochondria and phosphorylates Parkin. Phosphorylated Parkin then marks mitochondrial proteins for degradation via ubiquitination [20,21,22]. In early stages, mitophagy helps maintain a consistent metabolic rate through the removal of excess or faulty mitochondria. Several factors point to this tumor-suppressive role: Firstly, one study found that mitophagy dysfunction leads to ROS accumulation, causing uncontrolled energy homeostasis [23]. It also pointed to Parkin’s importance in the mitophagy pathway, showing that the inhibition of Parkin can lead to oncogenesis. In another paper focused on mouse models, the knockout of Parkin induced hepatocellular carcinoma (HCC) development [24]. Parkin was also found to prevent cancer invasion by marking hypoxia-inducible factor 1-alpha (HIF-1α) for degradation [25]. On the other hand, developed cancer cells use mitophagy to survive stress conditions by recycling abnormal mitochondria, and, by this stage, Parkin adopts a tumor-promoting role, demonstrating overexpression in cancers such as melanoma, relative to normal skin tissue. Increased Parkin levels were found to lead to enhanced growth and metastasis, while the inhibition of Parkin led to their suppression. Another mitophagic factor with a complex involvement in tumor growth is BCL2/adenovirus E1B 19 kDa protein-interacting protein 3 (BNIP3) [26]. BNIP3 inhibition in triple-negative breast cancer enhanced ROS-mediated mitophagy, promoting metastasis [27], whereas BNIP3 overexpression in hepatocellular carcinoma induced a greater degree of mitophagy, suppressing metastasis [28].

## 3. Autophagy and Hormone Sorting

### 3.1. Degradation of Secretory Granules

In the 1950s, Novikoff found digestive enzymes, including phosphatases [29,30], nucleases [31], and esterases [32] packaged in animal liver cells, leading to the discovery of the lysosome. While the precise molecular mechanisms governing hormone storage remain incompletely elucidated, accumulating evidence suggests that post-translational regulation of hormone storage dosage, independent of transcriptional modulation, plays a significant role in physiological signaling. In the 1970s, direct fusion of secretory granules with lysosomes, resulting in lysosomal digestion of hormones in the vesicles (crinophagy or granulolysis), was morphologically observed upon starvation in nerve cells [33]. This phenomenon was subsequently observed in pancreatic beta cells in a more recent study [34]. For instance, immunogold electron microscopy on purified mouse islets using an insulin-peptide specific antibody and a Lysosomal-associated membrane protein 1 (LAMP1) antibody detected granule structures containing both insulin B-chain peptides and LAMP1, indicating the presence of crinophagy [34]. Cellular digestion was primarily thought to be a result of nutrient restriction; these days, we now know that various stimuli are able to activate autophagy through mTOR, AMPK, and so on. In the pituitary gland, crinophagy also targets secretory granules, thereby regulating hormone levels [35]. Dysfunctional crinophagy in these cells can lead to endocrine disorders, highlighting its importance in maintaining cellular homeostasis [36]. This process functions as a quality-control mechanism, ensuring appropriate intracellular levels of secretory granules and preventing the accumulation of unnecessary secretory products [37]. Then, turnover of SVs mainly seemed to depend on crinophagy and, to a lesser extent, on another type of digestive system, autophagy, in resting pancreatic beta cells [38]. In the report, Meda described the presence of autophagic vacuoles (namely, autophagosomes) that sequester SVs and cytoplasmic materials. Overall, directly or indirectly, SVs are involved in the lysosomal digestive system, governing quantitative and qualitative maintenance of intracellular molecules. In yeast, Coat Protein Complex II (COPII) vesicles are formed from the ER-Golgi intermediate compartment upon starvation and subsequently serve as LC3-II-coated membranes, a key component of autophagosomes [39]. The observation indicates that the secretory pathway shares an initial step of vesicle formation with autophagy [40].

### 3.2. Unconventional Hormone Secretion

Secretory autophagy (SA) represents a non-canonical secretory pathway that integrates autophagic machinery with the cellular secretory process. This mechanism, governed by autophagy-related (ATG) proteins and Small GTPases (Rabs), plays a critical role in diverse cellular functions, notably in tumorigenesis and the development of treatment resistance. SA facilitates the release of cellular constituents, including cytokines, hormones, organelles, and pathogens [41], into the extracellular space, circumventing lysosomal degradation and consequently impacting the tumor microenvironment (TME) [42]. More recently, the LC3-mediated transport of insulin granules medicated by RAB-37 has been reported [43]. The work demonstrated that insulin homeostasis is maintained by SA, as well as by the conventional secretory pathway in MIN6 cells and mice. Briefly, there may be a common, evolutionarily conserved sorting platform for hormones, chemokines, and other cellular proteins into lysosomes (degradation) or the extracellular space (secretion) through autophagic machinery [44].

The interplay between autophagy and exosome biogenesis and release is well-established. A key example is the formation of amphisomes, hybrid vesicles arising from the convergence of autophagic and endosomal pathways. Exosomes, crucial mediators of intercellular communication, facilitate the transfer of bioactive molecules, thereby influencing recipient cell behavior [45]. Autophagy further modulates secretory processes by controlling the maturation and trafficking of conventional secretory vesicles. Perturbations in autophagosome maturation can alter extracellular vesicle (EV) secretion and the release of autophagy cargo receptors, contributing to proteostasis maintenance during endolysosomal dysfunction [46]. Furthermore, autophagy inhibition can impact endosome-related secretory pathways, altering EV secretion and cargo composition. These observations carry significant implications for therapeutic interventions, particularly in cancer, where autophagy modulation can exert both pro-tumorigenic and anti-tumorigenic effects [47].

### 3.3. Dysfunction of SV Degradation

In endocrine tissues, such as the pituitary gland, autophagy selectively targets secretory granules, thereby regulating hormone levels. Dysfunctional autophagy in these cells can lead to endocrine disorders, highlighting its importance in maintaining cellular homeostasis [36]. Within pituitary tumors, autophagy exhibits a dual role in regulating peptide hormone production. It contributes to cellular homeostasis by controlling the maturation and secretion of these hormones through crinophagy, a specialized form of selective autophagy. Crinophagy involves the direct fusion of excess or obsolete secretory granules with lysosomes, forming crinosomes, where the granule contents are degraded. This process functions as a quality-control mechanism, ensuring appropriate intracellular levels of secretory granules and preventing the accumulation of unnecessary secretory products [37]. Dysregulation of crinophagy has been implicated in several human disorders, including pituitary tumors, where it may contribute to the modulation of hormone production and secretion. Specifically, in pituitary tumors, crinophagy can influence the secretion of peptide hormones, potentially contributing to the pathophysiology of these neoplasms. Furthermore, aberrant autophagy can contribute to tumor progression by altering the secretion profiles of peptide hormones and other secretory proteins, potentially impacting tumor growth and the manifestation of hormone-related symptoms.

## 4. Targeting Autophagy as a Therapeutic Approach for Pituitary Tumors

A line of evidence demonstrates the involvement of autophagy in cell growth in pituitary tumors (PTs) [48,49,50,51,52,53]. A study investigated macroautophagy in pituitary neuropathology with regards to Beclin1 and Microtubule-associated protein 1 light chain 3 (LC3) proteins [53], revealing that 60% of functioning pituitary tumors exhibited signs of macroautophagy, with higher Beclin1 and LC3 expression levels in macro-pituitary tumors compared to micro-pituitary tumors. These results suggest that Beclin1 and LC3 play important roles in regulating pituitary cell growth and the progression of functioning pituitary tumors. Additional molecules and intracellular systems, including Forkhead box P1 (FOXP1)-induced long noncoding RNA CLRN1-AS1 [49], endoplasmic reticulum (ER) stress [51], hypoxia-inducible factor 1α (HIF-1α) [52], and the AMPK- ULK1 pathway [54], are also involved in the regulation of autophagy that controls pituitary tumor growth and/or hormone production via autophagy.

Targeting autophagy in cancer therapy requires careful consideration of the cancer’s developmental stage and the degree of autophagy activation. Current investigations in pituitary tumor management explore both increasing and decreasing autophagy as potential treatments. Numerous studies document changes in autophagy activity caused by compounds that exhibit anti-tumor effects in pituitary tumors.

### 4.1. Enhanced Autophagic Activity

Previous studies have shown that several autophagy activator drugs lead to reduced pituitary tumor cell growth and/or hormone production. A study investigates the effect of a potent Bone morphogenetic protein 4 agonist, BMPSB4, on autophagy in corticotroph pituitary tumors associated with Cushing’s disease (CD) [55]. A low expression of BMP4 in human ACTH-secreting pituitary tumor tissues was identified, where BMPSB4 was found to induce cellular autophagy in the AtT20 cell line through a SMAD-dependent pathway, leading to tumor growth inhibition. The research highlights BMPSB4’s potential as a therapeutic agent for CD by promoting autophagy-mediated cell death and reducing tumor growth and hormone secretion. Another study examines the effect of dimethylamino-micheliolide (ACT001) on pituitary tumors, which was well tolerated and demonstrated anti-tumor effects in patients with malignant glioma in a phase 1 clinical trial. Treatment with ACT001 with multiple biological activities, including the inhibition of the PI3K/AKT pathway [56], induced apoptosis by activated autophagy in GH3 cells and MMQ cells via activating JNK and P38 phosphorylation by binding to MEK4 [57]. ACT001 also reverses resistance to the dopamine agonist, cabergoline (CAB), in GH3 cells by inhibiting the mTOR-signaling pathway, inducing cell death through autophagy [58]. Another study used AtT20 and xenograft tumor models in mice to demonstrate anti-cancer effects of celastrol [59], which was shown to inhibit cell proliferation and migration, induce apoptosis, and trigger autophagy by downregulating the AKT/mTOR-signaling pathway. The findings support celastrol as a potential therapeutic drug for ACTH-secreting pituitary tumors [60].

Although suppression of excess hormone production is crucial to treat patients with pituitary tumors, these studies did not investigate the effect of the drugs on hormone production [55,57,60]. One other study explored the sphingosine kinase 1 (SphK1)/sphingosine-1-phosphate (S1P)/S1P receptor 2 (S1PR2) (SPHK1/S1P/S1PR2)-signaling pathway in GH-secreting pituitary tumors and assessed the effectiveness of the S1PR2 inhibitor JTE-013 [61], which showed abnormal SPHK1/S1P signaling in human GH-secreting pituitary tumors. The knockdown of SPHK1 suppresses S1P-mediated cell proliferation in GH3 cells by promoting autophagy and apoptosis with inhibition of GH secretion, which suggests that SIPR2 may be a promising therapeutic target for GH-secreting pituitary tumors.

Treatment with the dopamine receptor D5 (DRD5) agonist SKF83959 induces autophagosome formation in MMQ and GH3 cells, promoting autophagy and blocking autophagic flux in GH3 cells [62]. SKF83959 also enhances lysosomal acidification while impairing lysosomal degradation, thereby obstructing autophagic flux and resulting in the accumulation of Sequestosome 1(SQSTM1)/p62 and LC3. Additionally, CQ, a late-stage inhibitor of autophagy, can further obstruct autophagic flux and enhance DRD5-mediated pituitary tumor cell growth suppression. Rosiglitazone, a synthetic peroxisome proliferator-activated receptor γ (PPARγ) ligand, was found to decrease GH production and promote apoptosis and autophagy in GH3 cells and primary GH-secreting pituitary tumor cells [63]. Rosiglitazone increased the expression of molecules involved in autophagic processes, such as Beclin 1, ATG5, and ATG7, and reduced p62 expression, suggesting the activation of autophagy. Another potential therapeutic drug is cyclosporine A (CsA), which increases the expression of autophagy markers LC3-I and LC3-II and elevates levels of autophagosomes in GH3 cells, as indicated by immunofluorescence staining of LC3 and Lamp2, leading to apoptotic or autophagic cell death [64].

Several studies assessed the correlation of pituitary tumor-targeting drugs with autophagic activity in pituitary tumors. The somatostatin analog (SSTA) octreotide, known to suppress the proliferation of cultured rat GH-producing pituitary tumor (GH3) cells at a concentration of 10 nM [65], was observed in a recent study to induce autophagy activation and enhances autophagic flux at a higher concentration of 100 nM without reducing cellular metabolic activity in the same cell line [66]. Another study evaluated the effect of preoperative SSTA treatment on apoptosis and autophagy proteins in patients with acromegaly. Preoperative SSTA treatment increased TUNEL, caspase-3, and ATG-5 immunopositivity [67], which is involved in the formation of autophagosomes [68], while decreasing survivin and beclin-1 immunopositivity, which interacts with protein molecules either to activate or to deactivate autophagocytosis [69]. The findings suggest that SRL treatment might induce apoptosis, modulate autophagy, and decrease cell proliferation in GH-secreting adenomas through enhancing gene expression of ATGs and apoptosis cross-talk.

Another study explores the cytotoxic mechanism of bromocriptine (BRC) in prolactinoma treatment, focusing on autophagy [70]. BRC treatment accelerated the conversion of LC3-I to LC3-II and decreased prolactin secretion. The study concludes that BRC-treated pituitary tumor cells primarily undergo autophagic cell death, offering insights into BRC’s effectiveness against prolactinoma. Another dopamine agonist, CAB, increases autophagosome formation but blocks autophagic flux, accumulating undigested autolysosomes and cell death [71]. This anti-tumor effect of CAB is enhanced by DEP domain-containing mechanistic target of rapamycin-interacting protein (DEPTOR) [72]. The anti-pituitary tumor effects of the drugs enhancing autophagic activity were summarized in Table 1.

### 4.2. Inhibited Autophagic Activity in Pituitary Tumors

In contrast to the previous paragraphs regarding promoting autophagy as a therapeutic approach, several studies suggest autophagy inhibition for pituitary tumors. Pharmacological autophagy inhibition is also shown to suppress hormone production and cell growth in pituitary tumor cells [48]. The expression of several autophagy-inducible genes in pituitary tumor cell lines was identified, and the suppression of basal autophagy using RNA interference targeting Tfe1 inhibited cell proliferation. Treatment with autophagy inhibitors such as bafilomycin A1, chloroquine (CQ), and monensin reduced cell proliferation and hormone production [48]. The combination of CQ and temozolomide (TMZ) demonstrated an additive effect on inhibiting cell proliferation, suggesting a novel approach for treating TMZ-resistant pituitary tumors [48]. A study investigates the combined effects of CAB and CQ on autophagy and cell growth suppression in pituitary tumors and other cancers. CQ is known to induce the formation of autophagosomes and autophagic cell death, but CAB, often used to treat prolactinomas, sometimes faces intolerance and ineffectiveness. The results reveal that combining CAB and CQ enhances the suppression of proliferation in rat pituitary tumor cells and primary human pituitary tumor cells by blocking normal autophagic cycles and inducing apoptosis, suggesting a promising therapeutic strategy for increasing treatment efficacy in pituitary tumors and potentially other cancers [50]. In a case report of a patient with dopamine agonist-resistant prolactinoma, normalization of prolactin levels and sexual function were achieved with a combination therapy of hydroxychloroquine, which is a metabolite of CQ, and cabergoline, suggesting a potential novel treatment strategy [73].

Another study showed that PPARγ agonist 15d-PGJ2 blocks autophagic flux by reducing expression of the major lysosomal enzyme LAMP-1, resulting in the accumulation of SQSTM1/p62, an autophagy substrate, as well as LC3-II in vivo models of prolactin-secreting PT [74]. SQSTM1/p62 relies on autophagy for its degradation, and, therefore, the accumulation of p62 serves as an indicator for impaired autophagic flux [75,76]. Furthermore, the treatment of prolactin-secreting PT with 15-dPGJ2 suppressed tumor growth and prolactin production. These findings demonstrate that impaired autophagy flux contributed to autophagy dysfunction, and the use of 15-dPGJ2 may be a potential PT treatment option. The anti-pituitary tumor effects of the drugs inhibiting autophagic activity were summarized in Table 2.

## 5. Interpretation of Autophagy Activity

Autophagy can lead to autophagic cell death. LC3 serves as a marker for autophagy, undergoing conversion from the cytosolic form, LC3-I, to the modified form, LC3-II, which localizes to autophagosomal membranes, indicating activated autophagy [77]. However, blocking the later stages of autophagy, such as the fusion of autophagosomes with lysosomes, can lead to an accumulation of LC3-II, as it is not being degraded, resulting in an increased LC3-I to LC3-II conversion ratio [78]. Autophagy inhibitors exhibit varying effects based on their mechanisms of action. Early-stage inhibitors, such as 3-Methyladenine (3-MA) and Wortmannin, impede the initiation of autophagy, thereby decreasing overall autophagy activity by preventing the formation of autophagosomes [79,80]. In contrast, late-stage inhibitors, including chloroquine (CQ) and Bafilomycin A1, disrupt autophagic flux by inhibiting the fusion of autophagosomes with lysosomes or impairing lysosomal function. This disruption results in the accumulation of autophagosomes and their cargo, reflecting decreased autophagic flux, leading to the accumulation of LC3-II [48]. Therefore, while early-stage inhibitors reduce overall autophagy activity, late-stage inhibitors specifically decrease autophagic flux. Rigorous scrutiny is required when interpreting findings on autophagic activities and autophagic flux modulated by reagents that are not autophagy inhibitors. Furthermore, as suggested by Mizushima and Murphy, precise autophagy flux determination requires a multi-faceted approach, integrating fluorescent/pH reporters with mechanism-based readouts, as no single assay is conclusive [81].

While prior investigations have established the participation of autophagy in secretory pathways described in the section “Autophagy and hormone sorting”, the precise impact of autophagy inhibition on hormone synthesis and release within pituitary tumors remains relatively unknown [74]. The molecular mechanisms through which autophagy blockade attenuates hormone production in pituitary tumor cells are not fully elucidated. However, due to the established role of autophagy in the lysosomal degradation of intracellular proteins into constituent amino acids, and the subsequent translocation of these amino acids to the endoplasmic reticulum (ER) for protein synthesis, including peptide hormones, a plausible hypothesis emerges. Specifically, it is proposed that the pharmacological suppression of autophagy results in a diminution of available amino acid pools within the ER lumen, thereby limiting the substrate availability for pituitary hormone synthesis. Furthermore, a concomitant reduction in the proliferative capacity or viability of hormone-producing pituitary tumor cells may contribute to the observed decrease in hormone hypersecretion. Collectively, these observations suggest that targeted modulation of autophagy pathways represents a potential therapeutic strategy for the management of hormone excess in pituitary tumorigenesis.

## 6. Limitations of Current Models

A significant discrepancy exists in reported hormonal outcomes following autophagy modulation, with studies employing inhibitory compounds consistently demonstrating suppressed hormone production, while those utilizing autophagy-promoting agents exhibit less uniform and comparatively limited results. Prior investigations have elucidated the dualistic roles of autophagy in pituitary tumor cells, revealing both inhibitory and stimulatory influences on hormone synthesis and secretion (Table 1 and Table 2). This apparent paradox underscores the context-dependent and multifaceted nature of autophagy’s involvement in cellular physiology, influenced by factors including the cellular milieu, prevailing signaling cascades, and the specific stage of tumor progression. Further rigorous investigation into the underlying molecular mechanisms governing this functional duality is imperative for the rational design of therapeutic strategies targeting autophagy in pituitary tumors.

Rat and mouse pituitary tumor cell lines, including the most commonly used GH3 cells and AtT20 cells, being derived from single tumors, have a limited ability to represent the complexity of human pituitary tumors, as well as their diverse cellular compositions and genetic profiles. The simplified microenvironment of cell lines lacks the three-dimensional architecture and extracellular matrix interactions found in native tumors, such as vascularization or immune system interactions. These may influence cell behavior and drug response. Some studies explore the potential of 3D cell culture models, such as organoids, to better mimic the native tumor microenvironment [82]. Cell lines can also undergo genetic and epigenetic changes as they develop, which may lead to changes in native tumor characteristics and behavior. These discrepancies may be mediated by further development of human pituitary tumor cell lines or induced pluripotent stem cell-derived pituitary cells to more closely simulate the in vivo environment of human pituitary tumors.

## 7. Clinical Perspective

Targeting autophagy presents a complex yet potentially valuable strategy for pituitary tumor management. Preclinical data suggest autophagy inhibition can suppress tumor growth and hormone hypersecretion by inducing apoptosis and disrupting secretory pathways. However, clinical translation is hindered by tumor heterogeneity, the pleiotropic roles of autophagy, and the limited availability of FDA-approved inhibitors, primarily chloroquine and hydroxychloroquine. While effective in other cancers, their application in pituitary tumors remains nascent, with concerns regarding off-target toxicity and resistance. Robust clinical trials are essential to determine optimal dosing, patient selection, and long-term safety. Future research should focus on identifying predictive autophagy-related biomarkers to facilitate personalized therapy.

## 8. Conclusions

**Figure 1:**
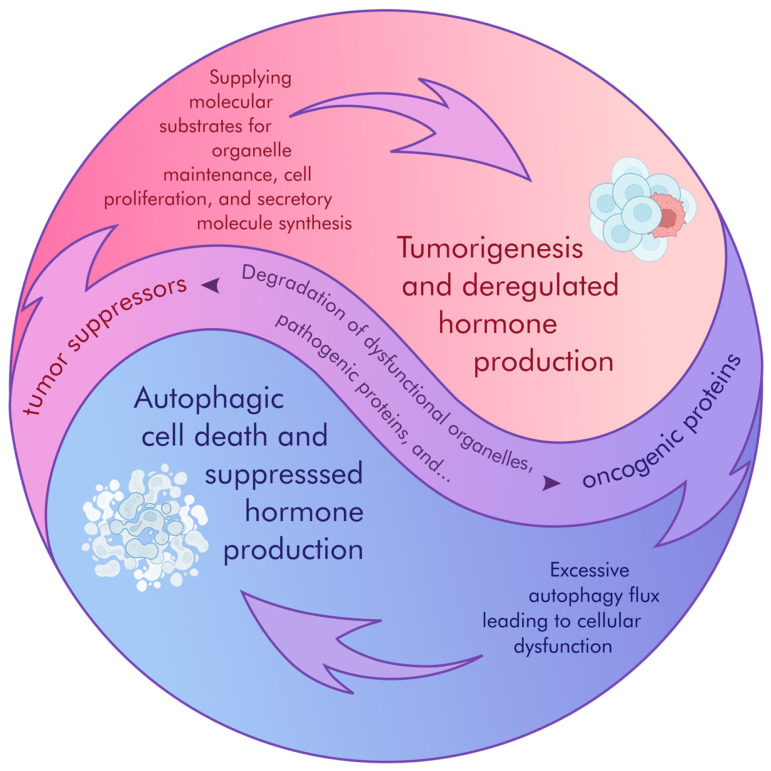
Double-role autophagy in pituitary tumor pathogenesis. Autophagy-mediated pituitary tumorigenesis: Under specific conditions, autophagy can facilitate pituitary tumor growth and hormone production. Mechanisms include: supplying amino acids and metabolic substrates through the degradation of cellular components, supporting increased energy demands of rapidly proliferating tumor cells, enhancing signals for cellular proliferation and synthesis of secretory molecules, including peptide hormones. Autophagy-mediated pituitary tumor suppression: Conversely, autophagy can also exert tumor-suppressive effects. This can occur through: selective degradation of oncogenic proteins or damaged organelles, preventing their accumulation and mitigating pro-tumorigenic signaling, sustaining signals for cellular proliferation and synthesis of secretory molecules, leading to decreased hormone secretion, and potentially limiting tumor growth. Otherwise, excessive or unrestricted autophagy leads to cell death. The balance between these opposing functions of autophagy is likely determined by the specific genetic and microenvironmental context of the pituitary tumor.

Autophagy activation can drive pituitary tumor cell proliferation or trigger cell death. Some hypotheses might explain this duality (Figure 1). When pituitary tumor cells are under stress, such as medical treatment, autophagy can be activated to promote cell survival by repairing and restructuring the cells. In this case, autophagy supports cell proliferation. On the other hand, excessive activation of autophagy can cause significant damage to the cell structure and function, leading to cell death. In this scenario, autophagy induces cell death. Thus, the activation of autophagy can have different effects depending on the state and environment of the cells. This duality can be explained by the levels of cellular stress and the mechanisms that regulate autophagy. Furthermore, as autophagy is involved in the secretory process, targeting autophagy would be promising to regulate hormone production in pituitary tumors. Ultimately, the long-term impact of anti-tumor effects by selected drugs remains unclear, and further studies are required to select between inhibiting or promoting autophagy in pituitary tumors. Clinical trials and further research are essential to determine the most effective approach for each individual case.

## Figures and Tables

**Table 1:** Drugs enhancing autophagic activity in pituitary tumors.

Enhanced Autophagic Activity				
Drug (Concentration/Dose)	Target (Species)	Effect on Cell Proliferation	Effect on Hormone Production/Secretion	Reference Number
BMPSB4 (39.05 μM ^a^)	AtT20 (m)	Suppressed	NA	[55]
ACT001 (9.56 μM ^a^, 22.65 μM ^a^, 20 μM ^b^)	GH3 (r), MMQ (r), NFPT (h)	Suppressed	NA	[57,58]
Celastrol (1 μM ^a^)	AtT20 (m)	Suppressed	NA	[60]
JTE-013 (41.17 μM ^a^)	GH3 (r)	Suppressed	GH suppressed	[61]
SKF83959 (5 μM ^b^)	GH3 (r), MMQ (r)	Suppressed	NA	[62]
Rosiglitazone (50 μM ^b^)	GH3 (r)	Suppressed	GH suppressed	[63]
CsA (0.1 μM ^b^)	GH3 (r)	Suppressed	NA	[64]
Octreotide (100 nM ^c^)	GH3 (r)	NA	NA	[66]
BRC (110 μM ^a^, 60 μM ^a^)	GH3 (r), MMQ (r)	Suppressed	PRL suppressed	[70]
CAB (100 μM ^a^, 50 μM ^a^)	GH3 (r), MMQ (r)	Suppressed	NA	[71]

^a^ IC50: half-maximal inhibitory concentration, ^b^ LIC: lowest inhibiting concentration, ^c^ tested concentration, CsA: cyclosporine A, BRC: bromocriptine, CAB: cabergoline, NFPT: non-functioning pituitary tumor, GH: growth hormone, PRL: prolactin, NA: not assessed, m: mouse, r: rat, and h: human.

**Table 2:** Drugs inhibiting autophagic activity in pituitary tumors.

Inhibited Autophagic Activity				
Drug (Concentration/Dose)	Target (Species)	Effect on Cell Proliferation	Effect on Hormone Production/Secretion	Reference Number
CQ (5 μM ^b^, 10 μM ^b^)	AtT20 (m), GH4 (r)	Suppressed	ACTH and GH suppressed	[48]
bafilomycin A1 (1 nM ^b^)	AtT20 (m)	Suppressed	ACTH suppressed	[48]
Monensin (2.5 μM ^b^)	AtT20 (m)	Suppressed	ACTH suppressed	[48]
CQ + TMZ (5 μM ^b^ + 20 μM ^b^, 10 μM ^b^ + 400 μM ^b^)	AtT20 (m), GH4 (r)	Suppressed	ACTH and GH suppressed	[48]
CQ + CAB (20 μM ^c^ + 100 μM ^c^, 20 μM ^c^ + 50 μM ^c^, 20 μM ^c^ + 50 or 100 μM ^c^)	GH3 (r), MMQ (r), Prolactinoma (h)	Suppressed	PRL suppressed	[50]
HCQ (200 mg/day ^c^) + CAB (3 mg/week ^c^)	Prolactinoma (h)	No recurrence	PRL suppressed	[73]
15d-PGJ2 (4 μg/day ^c^)	Prolactinoma (r)	Suppressed	PRL suppressed	[74]

^b^ LIC: lowest inhibiting concentration, ^c^ reported concentration/dose, CQ: chloroquine, TMZ: temozolomide, CAB: cabergoline, ACTH: adrenocorticotropic hormone, GH: growth hormone, PRL: prolactin, m: mouse, r: rat, and h: human.
